# Life in the Current: Anatomy and Morphology of *Utricularia neottioides*

**DOI:** 10.3390/ijms21124474

**Published:** 2020-06-23

**Authors:** Bartosz J. Płachno, Lubomír Adamec, Piotr Świątek, Małgorzata Kapusta, Vitor F. O. Miranda

**Affiliations:** 1 Department of Plant Cytology and Embryology, Institute of Botany, Faculty of Biology, Jagiellonian University in Kraków, 9 Gronostajowa St., 30-387 Cracow, Poland; 2 Institute of Botany CAS, Department of Experimental and Functional Morphology, Dukelská 135, CZ-379 01 Třeboň, Czech Republic; lubomir.adamec@ibot.cas.cz; 3 Institute of Biology, Biotechnology and Environmental Protection, Faculty of Natural Sciences, University of Silesia in Katowice, 9 Bankowa St., 40-007 Katowice, Poland; piotr.swiatek@us.edu.pl; 4 Department of Plant Cytology and Embryology, University of Gdańsk, ul. Wita Stwosza 59, 80-308 Gdańsk, Poland; malgorzata.kapusta@ug.edu.pl; 5 Departamento de Biologia Aplicada à Agropecuária, Universidade Estadual Paulista (Unesp), Faculdade de Ciências Agrárias e Veterinárias, Jaboticabal, SP CEP 14884-900, Brazil; vitor.miranda@unesp.br

**Keywords:** aquatic plants, carnivorous plants, Cerrado, Lentibulariaceae, plant anatomy, rheophytes, cell-wall components

## Abstract

Rheophytism is extremely rare in the *Utricularia* genus (there are four strictly rheophytic species out of a total of about 260). *Utricularia neottioides* is an aquatic rheophytic species exclusively growing attached to bedrocks in the South American streams. *Utricularia neottioides* was considered to be trap-free by some authors, suggesting that it had given up carnivory due to its specific habitat. Our aim was to compare the anatomy of rheophytic *U. neottioides* with an aquatic *Utricularia* species with a typical linear monomorphic shoot from the section *Utricularia*, *U. reflexa*, which grows in standing or very slowly streaming African waters. Additionally, we compared the immunodetection of cell wall components of both species. Light microscopy, histochemistry, scanning, and transmission electron microscopy were used to address our aims. In *U. neottioides*, two organ systems can be distinguished: organs (stolons, inflorescence stalk) which possess sclerenchyma and are thus resistant to water currents, and organs without sclerenchyma (leaf-like shoots), which are submissive to the water streaming/movement. Due to life in the turbulent habitat, *U. neottioides* evolved specific characters including an anchor system with stolons, which have asymmetric structures, sclerenchyma and they form adhesive trichomes on the ventral side. This anchor stolon system performs additional multiple functions including photosynthesis, nutrient storage, vegetative reproduction. In contrast with typical aquatic *Utricularia* species from the section *Utricularia* growing in standing waters, *U. neottioides* stems have a well-developed sclerenchyma system lacking large gas spaces. Plants produce numerous traps, so they should still be treated as a fully carnivorous plant.

## 1. Introduction

The small generic section *Avesicaria* of the genus *Utricularia* L. (bladderwort) contains only two South American aquatic or partly amphibious, rheophytic species: *Utricularia neottioides* A.St.-Hil. & Girard and *U. oliveriana* Steyerm [1,2,3]. These exclusively grow attached to rocks in shallow streaming or seeping waters While the latter species closely resembles the typical, smaller terrestrial *Utricularia* species with spatulate leaves, *U. neottioides*, is morphologically one of the most modified and remarkable *Utricularia* species. It is specialized for growth in fast-flowing waters by forming two types of shoots (stolons; [1,4,5] and the literature therein); attached anchor stolons (claw-like, radiate ‘rhizoids’), which fix the plant to the rocky substrate by adhesive rhizoids, and long (dozens of cm) running stolons flowing freely in the streams and bearing 1–4 cm long filamentous “leaves” (in fact they are modified leaf-like shoots; see e.g., [6]). The “leaves” are only ca. 60 µm thick, densely covered by colorless hydrophobic trichomes (hairs) and they are reminiscent of filamentous algae [4]. The inflorescence scape growing from anchor stolons may be up to 30 cm long and bears several whitish and sweet-scented flowers. The pollinators of *U. neottioides* remain a mystery, but it is possible that small insects are attracted by the soft and sweet fragrance of the opened flowers (this subject is under study by our research group). Long discussions have been conducted on the abundance of traps in this species as only a few traps (if any) have been collected from the field sites and found in herbaria (see [1,4]). Moreover, no single trap was produced either in the aseptic in vitro culture or in terrestrially grown plants on brown peat [4]. Rheophytes are plants usually confined and adapted to streambeds and/or below the level of flooding and the plants can be subject to temporary overflowing [7]. Rheophytism is rare in *Utricularia* and is represented by a few species from different sections, including sect. *Avesicaria*. Following the phylogenetic evidence, the rheophytism appeared at least twice in the evolutionary history of *Utricularia* [5,8,9] as it results from the homoplastic processes of parallel evolution with terrestrial species as an ancestral form.

In contrast with the rheophytic *U. neottioides*, typical aquatic *Utricularia* species from the section *Utricularia* grow in standing or very slowly streaming waters (e.g., *U. australis*, *U. foliosa*, *U. reflexa*) and possess a quite different habit [10]. They have a linear, modular shoot structure which consists of leaf nodes with finely pinnate, filamentous leaves and narrow tubular internodes [1,11,12]. The majority of species with linear shoots have monomorphic (homogeneous, non-differentiated) green shoots bearing traps. However, several species have dimorphic shoots differentiated into pale carnivorous ones with all or the majority of traps and green photosynthetic ones without traps or with only few traps. All aquatic *Utricularia* species with linear shoots form regular branches which allows rapid propagation. Moreover, adult individuals typically show very rapid apical shoot growth of 1–4.2 new leaf nodes per day but their basal shoot segments die at about the same rate. Very rapid apical shoot growth with frequent shoot branching underlie the total, very high relative growth rate [11,12]. 

After the terrestrial life form, the aquatic is the most common of all *Utricularia* species [13]. The aquatic species can be classified in different subtypes, as suspended (freely) or affixed forms, occurring in several sections, but most species are from sect. *Utricularia* [1,9]. Based on phylogenetic hypotheses, the aquatic lineages are derived from the terrestrial ones through different events within the genus *Utricularia* [5,9]. 

Aquatic *Utricularia* species usually grow in shallow standing or slowly streaming humic, oligo-mesotrophic waters and a partly decomposed, nutrient-poor organic sediment (sedge or reed litter, peat, or fen substrate) usually accumulates on the bottom in these waters [11,12]. The waters are usually poor in minerals such as N, P, and sometimes also K, but enriched in free CO_2_ mostly within 0.1–1 mM which supports rapid plant growth. The species can usually tolerate higher total concentration of humic acids and tannins up to 60 mg/L. The waters are usually not saturated by oxygen. Most aquatic *Utricularia* species grow in soft to moderately hard, acid, or neutral waters (usually pH 5.7–7.0) but some temperate species can also occur in hard and alkaline waters [11,12]. 

*Utricularia neottioides* is distributed across a relatively large territory of tropical South America—in Colombia, Venezuela, Bolivia, and mainly in Brazil [1,3]. The plants can form large stands in suitable habitats with only inflorescences emerging above the water surface, but the stands are usually monospecific, without other co-occurring higher species [4]. In permanent shallow streams or in separated pools after a water-level decline, the plant can grow as a perennial, while in dried-out streams during the dry season, as an annual, usually setting seeds for the next season. The plant can also survive in temporary pools with standing water. As specified by Rivadavia [14] typical habitats are shallow, swiftly flowing, cool, acidic, humic (brownish) streams of highland areas from ca. 300–1800 m a.s.l. and the plants usually grow under bright sunshine.

The plant grows very vigorously and rapidly in a sterile in vitro culture in a half-strength Gamborg B5 medium with 2.5% sucrose [4]. The plant formed very thin stolons with finely filamentous leaves 2–5 cm long. However, any growth ex vitro as submerged in humic waters in aquaria was impossible for unknown reasons. The plant could, however, rapidly grow in a terrestrial form with 8–18 mm long filamentous leaves (diam. ca. 100–120 µm) which survived on the brown peat for 5–6 months but did not propagate further [4]. 

The main aim of the present paper is to characterize in detail the shoot morphology and anatomy of rheophytic *U. neottiodes*. Additionally, we compare it with those of a typical member of the section *Utricularia*, namely aquatic *U. reflexa* Oliv., a species with linear monomorphic shoots adapted to growing in standing waters in Africa. We also performed immunodetection of cell-wall components to determine if there were any differences between *U. neottiodes* and *U. reflexa*.

## 2. Results

### 2.1. Utricularia neottioides

**Figure 1:**
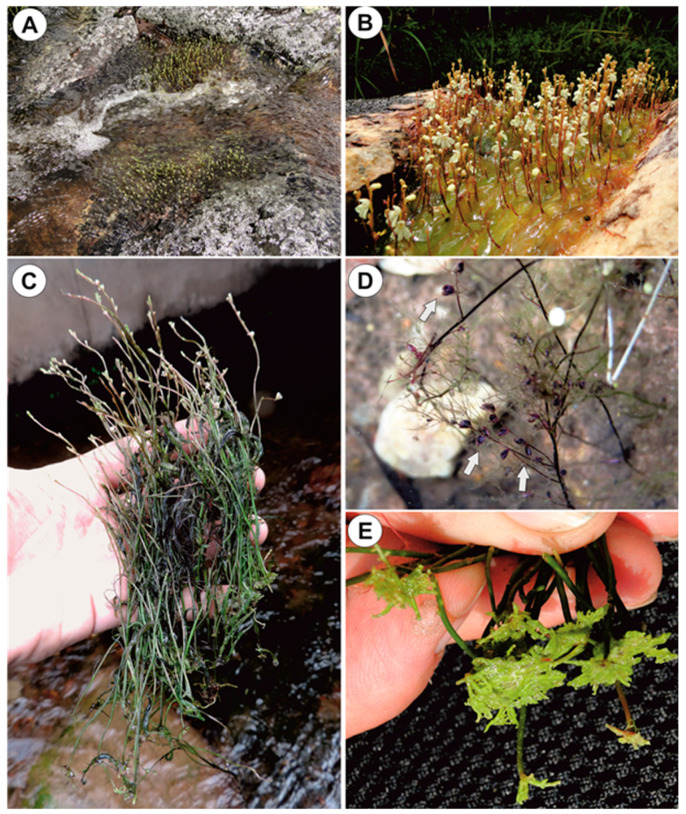
Habitat and general morphology of *U. neottioides*. (**A**,**B**) *Utricularia neottioides* in a natural habitat, the Serra da Canastra, southern Minas Gerais State (southeastern Brazil). (**C**) Exposed plants. (**D**) Leaf-like shoots with traps (white arrows). (**E**) Anchor systems of plants removed from rocks.

**Figure 2:**
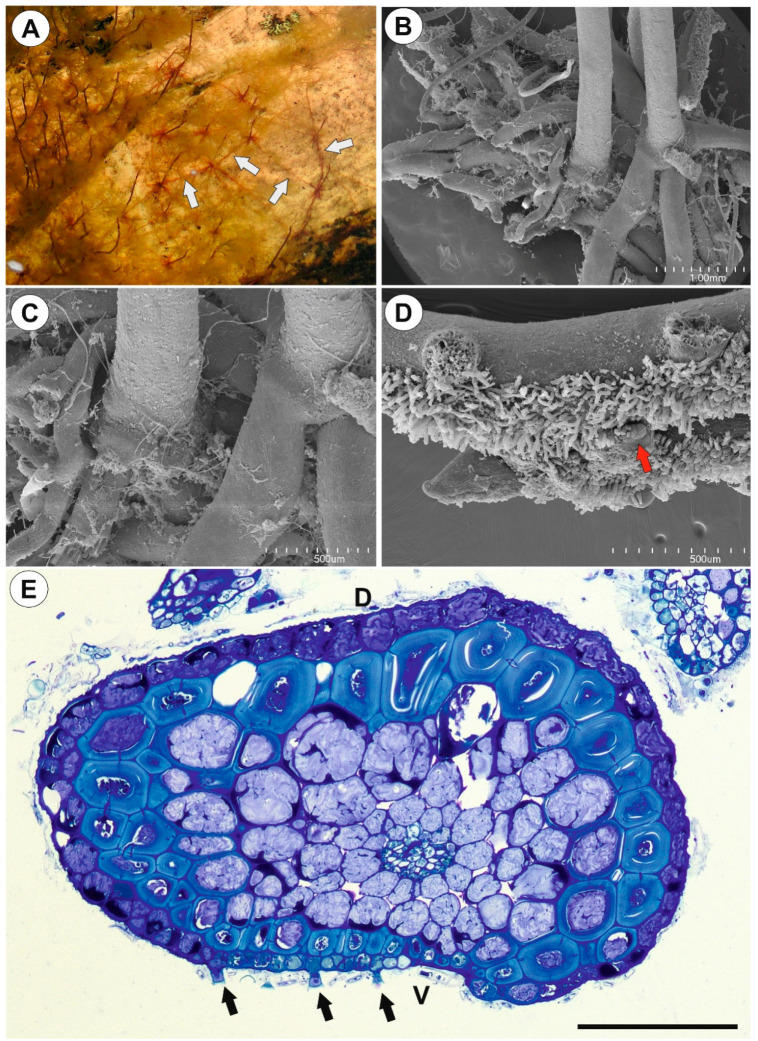
Anchor system structure of *U. neottioides.* (**A**) *U. neottioides* growing at rock surface; note running stolons (white arrows), which are used for vegetative reproduction of this species. (**B**,**C**) Basal portion of inflorescence stalk with claw-like anchor stolons, bar 1 mm (**B**) and 500 µm (**C**). (**D**) Anchor stolons; note adhesive hairs on the ventral side of the stolons and formation of new stolons (red arrow) on the ventral-lateral side of the stolon, bar 500 µm. (**E**) Transversal anchor stolon section, note adhesive trichomes (black arrows), D-dorsal side, V-ventral side, bar 100 µm.

The plant body consists of (Figure 1A–E): anchor stolons (fixing plants to rock, Figure 1E and Figure 2A–D), running stolons, which produce new rosettes (Figure 2B), stolons bearing leaf-like shoots and forming an inflorescence. Traps occur both on anchor stolons and leaf-like shoots.

#### 2.1.1. Anchor Stolons (Claw-Like ‘Rhizoids’) Fixing Plant

**Figure 3:**
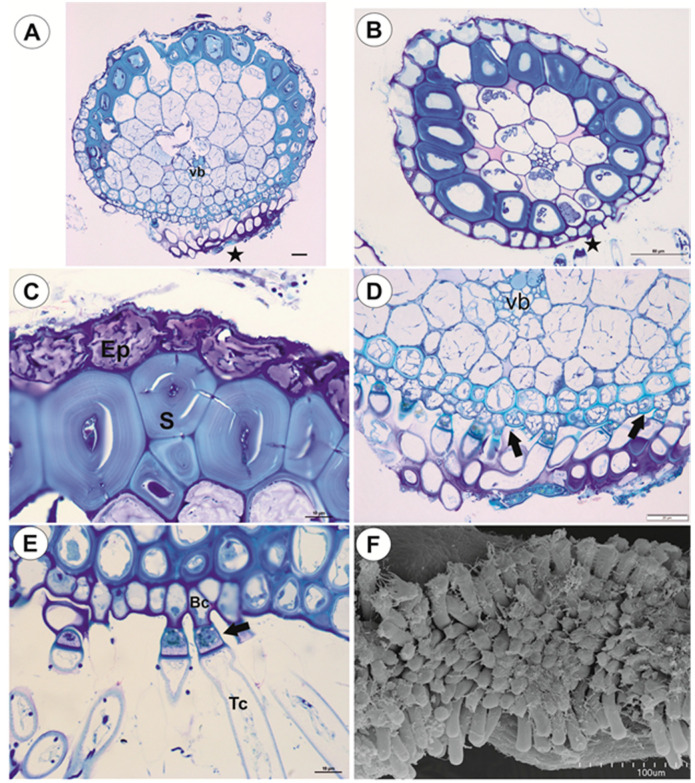
Structure of anchor stolons of *U. neottioides.* (**A**,**B**) Transversal anchor stolon section, note adhesive trichomes (stars), vascular bundle (vb), bar 20 µm (**A**) and 50 µm (**B**). (**C**) Dorsal part of anchor stolon; note epidermal cells contain mucilage in vacuoles (Ep) and sclerenchyma (S), bar 10 µm. (**D**) Ventral part of anchor stolon; note lignification of cell walls of some epidermal cells (arrows), vascular bundle (vb). Secretion produced by trichomes stain positively with MB/AII; note also microorganisms attached to secretion, bar 20 µm. (**E**) Structure of adhesive trichomes: basal cell (Bc), pedestal cell (arrow), and a head cell (Tc), bar 10 µm. (**F**) Morphology of adhesive trichomes; note remains of secretion, bar 100 µm.

**Figure 4:**
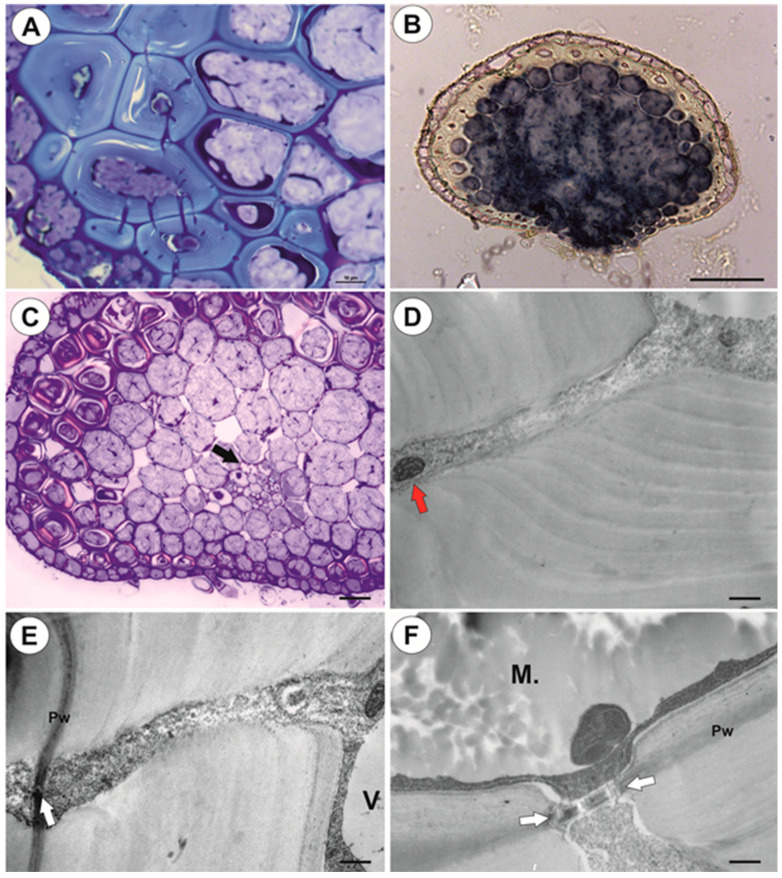
Anatomy of anchor stolons of *U. neottioides.* (**A**) Pits in cell walls of sclerenchyma cells, bar 10 µm. (**B**) Transversal section of anchor stolon treated with zinc iodine chloride solution, bar 100 µm. (**C**) Lignificated cell walls of sclerenchyma cells and vessels (black arrow) reflect polarized light, bar 27 µm. (**D**–**F**) Ultrastructure of pits of sclerenchyma cells: mitochondrion in cytoplasmic canal (red arrow), primary wall (Pw), plasmodesmata (white arrow), vacuole (V), mucilage in vacuole (M), bar 0.5 µm (**D**), 0.4 µm (**E**), and 0.6 µm (**F**).

**Figure 5:**
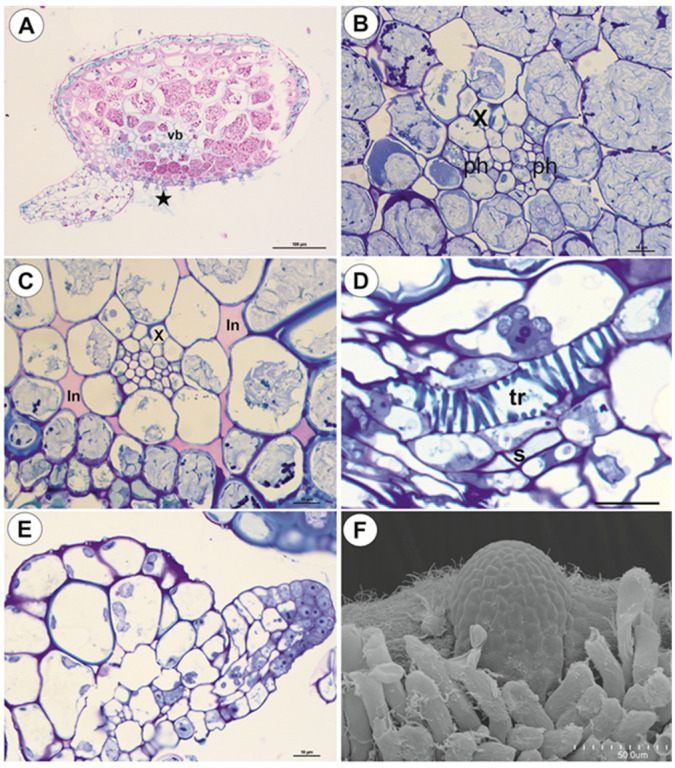
Anatomy of anchor stolons of *U. neottioides.* (**A**) Transversal section of anchor stolon, PAS reaction, vascular bundle (vb), adhesive trichomes (star), bar 100 µm. (**B**–**D**) vascular bundle structure: xylem (x), phloem (ph), intercellular spaces (in), tracheary element–vessel (tr), sieve tube (S), all bars 10 µm. (**E**,**F**) Formation of new stolon on the ventral-lateral side of the stolon, bar 10 µm (**E**) and 50 µm (**F**).

The organ has an asymmetric structure and is flattened ventrally (lower side, Figure 2D,E), with the parenchyma being better developed on the dorsal and lateral sides than on the ventral side. Epidermal adhesive trichomes (produce substances which glue the stolon to rocks) occur along the ventral side (Figure 2D,E) and the epidermal cells vary in size depending on their location. Cells on the ventral side are much smaller than those on the dorsal side (Figure 2E and Figure 3A,B). Cuticle has folds and these folds are not only composed of a cuticle but represent an irregular cell-wall contour covered by a cuticle (Figure 3C). The cell walls of some epidermal cells are lignified, especially on the organ’s ventral side. Epidermal cells have chloroplasts (Supplementary Materials Figure S1A) and contain mucilage-like material in vacuoles. This material positively stained with MB/AII (Figure 2E and Figure 3C) does not stain positively with ruthenium red (Supplementary Materials Figure S2A). Starch grains occur (Supplementary Materials Figure S2C). Adhesive trichomes occur only along the ventral side (Figure 3A,B,D), each consisting of one bottle-shaped basal cell, one short, pedestal cell and a head cell (Figure 3E). The lateral wall of the pedestal cell is impregnated by cutin and the head cell is strongly elongated. They produce a secretion (Figure 3F), which is not PAS positive but stains positively with MB/AII (Figure 3D) and the head cells stain positively with ruthenium red. Beneath the epidermis, there is a hypodermis containing sclerotic cells. The sclerenchyma forms a sheath, which surrounds a ground tissue and a cortex The stolon hypodermis has up to three layers (Figure 2E and Figure 3A,B), with the number positively dependent on stolon size. Sclerenchyma cells are the most well-developed on the dorsal and lateral sides. The cells retain their protoplasts, contain chloroplasts, and mucilage-like material (Figure 4A,F; Supplementary Materials Figure S1B,C). Cell walls of the sclerenchyma cells are thick with highly visible layers and are lignified (Figure 4A,D–F). They stain yellow with zinc iodine chloride solution (Figure 4B) and they reflect polarized light (Figure 4C). Pits occur and some of these are branched (Figure 4A). The cytoplasm strand of the pits can include mitochondria (Figure 4D). Parenchyma cells contain strongly-staining cytoplasm and are mostly filled with a PAS-positive material (probably mucilage) (Figure 5A,B). This material stains slightly with Lugol (Supplementary Materials Figure S2). However, this material does not stain positively with ruthenium red (Supplementary Materials Figure S2A). Starch grains occur (Supplementary Materials Figure S2B,C). There are small intercellular spaces between the parenchyma cells (Figure 5B,C), sometimes filled with a material, which stains pink with MB/AII (Figure 5C). The vascular bundle is small and is situated closer to the ventral side and it consists of both xylem and phloem; the xylem occurs on the upper part of the bundle (Figure 5B). One or two tracheary elements occur. Vessels have reticulate pits (Figure 5D) and the vessel diameter varies from 7.5–9 μm. The phloem is better developed than the xylem and sieve tubes are well visible (Figure 5D). Formation of new stolons (branching) is observed on the ventral-lateral side or the ventral side (Figure 5E,F). 

#### 2.1.2. Running Stolons

**Figure 6:**
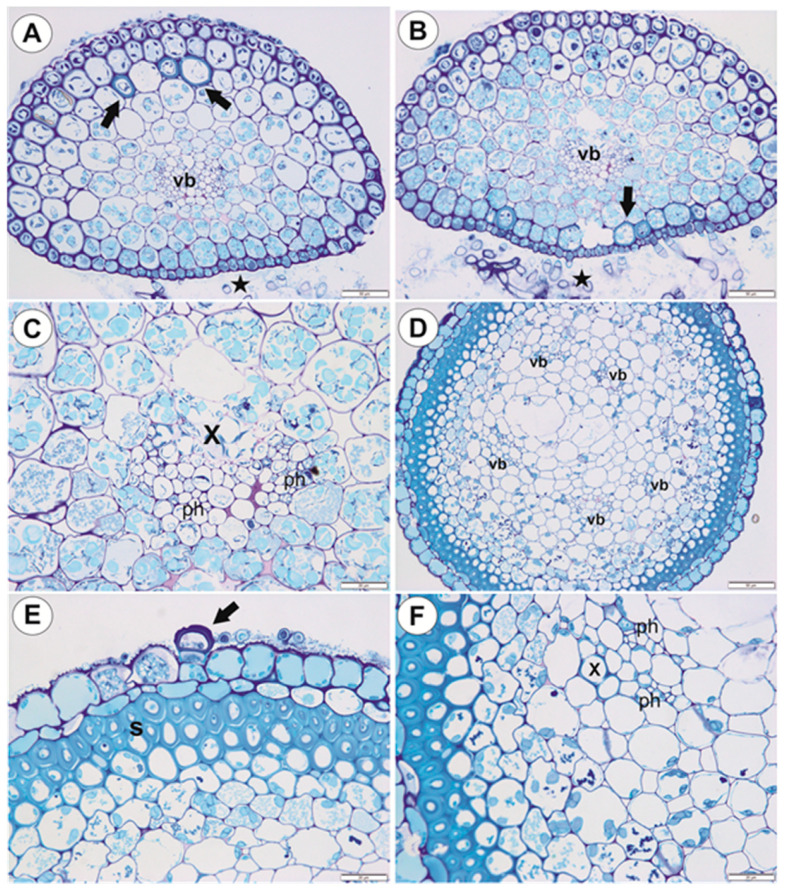
Anatomy of running stolons and inflorescence stalk of *U. neottioides.* (**A**,**B**) Transversal running stolon section; note adhesive trichomes (stars), vascular bundle (vb), sclerenchyma cells (arrows), bars 50 µm. (**C**) Vascular bundle in the running stolon: xylem (x), phloem (ph); note starch grains in parenchyma cells, bar 20 µm. (**D**–**F**) Transversal section of inflorescence stalk: vascular bundle (vb), sclerenchyma (S), epidermal trichome (arrow), phloem (ph), xylem (x), bar 50 µm (**D**) and 20 µm (**E**,**F**).

Running stolons have a similar structure to anchor stolons (Figure 6A–C). However, the difference occurs in the sclerenchyma development. There are only a few sclerenchyma cells on the dorsal side (Figure 6A) or only on the ventral side (Figure 6B). Epidermal adhesive trichomes occur along the ventral side (Figure 6A,B). In thicker stolons, xylem is better developed than in thinner ones, and up to seven vessels may occur (Figure 6C).

#### 2.1.3. Basal Portion of Inflorescence Stalk-Stolon Bearing Leaf-Like Shoots and Forming Inflorescence

As shown in the transverse sections, the organ is round (Figure 6D) and there is no clear border between the cortex and the pith. Beneath the epidermis, there is a one-layered parenchyma and then the sclerenchyma, which forms a continuous lignified fiber belt (Figure 6D,E) with about five small vascular bundles (Figure 6D). The xylem and the phloem are readily visible. In the vascular bundle, the xylem occurs more to the outside than the phloem (Figure 6F). The cuticle of epidermal cells has folds. The epidermal cells have chloroplasts (Figure 6E) and there are epidermal trichomes: each trichome consists of basal cell, barrier cell, and terminal cell (Figure 6E). Parenchyma cells contain starch grains (Figure 6F).

#### 2.1.4. Filamentous Leaf-Like Shoots

**Figure 7:**
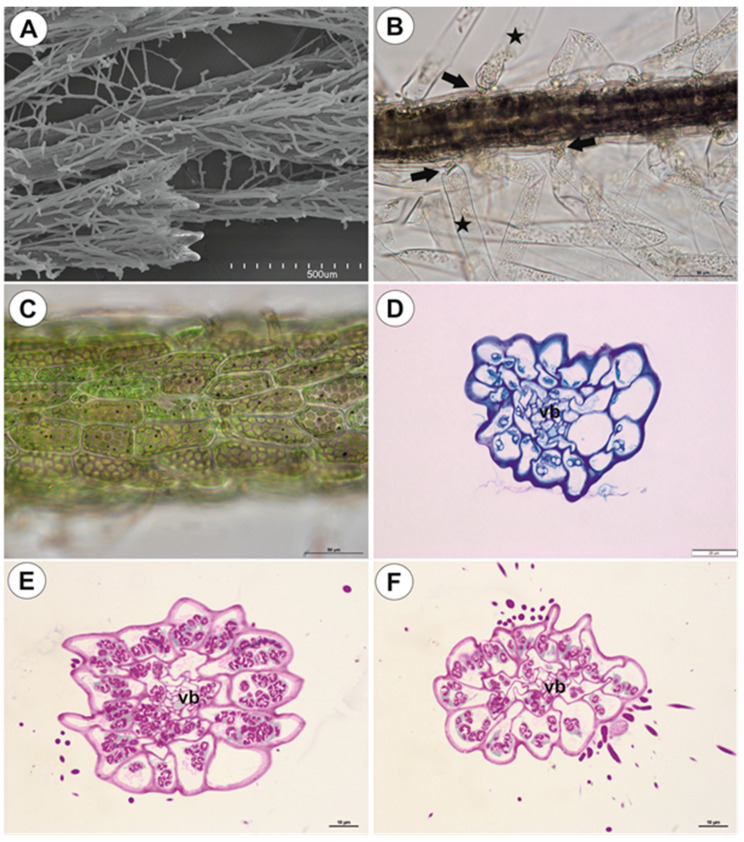
Structure of leaf-like shoots of *U. neottioides.* (**A**) Morphology of shoots; note that they are densely covered by epidermal trichomes, bar 500 µm. (**B**) Structure of epidermal trichomes: pedestal cell (arrow) and a head cell (star), bar 50 µm. (**C**) Chloroplasts in epidermal cells of shoot; note dark starch grains (after Lugol’s staining), bar 50 µm. (**D**) Transversal shoot section: vascular bundle (vb), bar 20 µm. (**E**,**F**) Transversal shoot section, after PAS reaction; note numerous starch grains: vascular bundle (vb), both bars 10 µm.

Leaf-like shoots branch dichotomously, exhibit apical growth (Figure 7A), and they bear traps (Figure 1D). However, traps may also occur on the anchor stolons. The surface of the shoots is covered by numerous trichomes (Figure 7A,B): each trichome consists of a basal cell, a barrier cell, and an elongate terminal cell (Figure 7B). Epidermal cells have chloroplasts and amyloplasts (Figure 7C–F). Filamentous leaf-like shoots have very simple structure; there is an epidermis, one or two layers of parenchyma and a centrally located vascular bundle (Figure 7D–F). In smaller shoots, the phloem occurs but vessels are not observed in the vascular bundle.

#### 2.1.5. Immunodetection of Cell Wall Components

**Figure 8:**
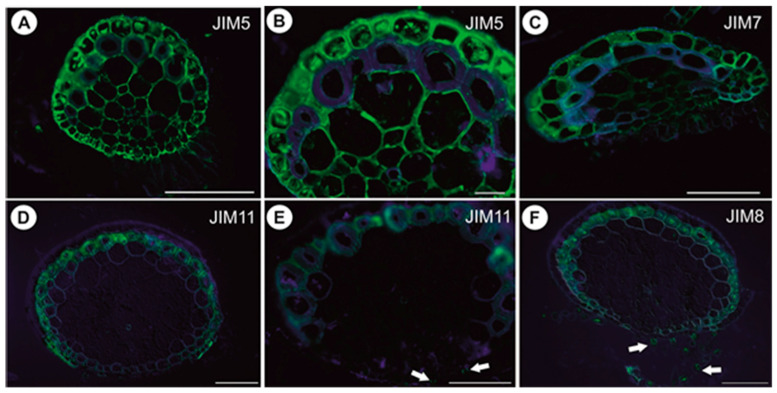
Pectin (JIM5, JIM7), extensin (JIM11) and arabinogalactan protein (JIM8) detection in anchor stolons of U. neottioides. (**A**,**B**) Presence of the JIM5, bar 100 µm (**A**) and 20 µm (**B**). (**C**) Presence of the JIM7, bar 100 µm. (**D**,**E**) Presence of the extensin; note positive signal in cell walls of barrier cells of trichomes (arrows), both bars 100 µm. (**F**) Presence of the JIM8; note positive signal in trichomes (arrows), bar 100 µm.

**Figure 9:**
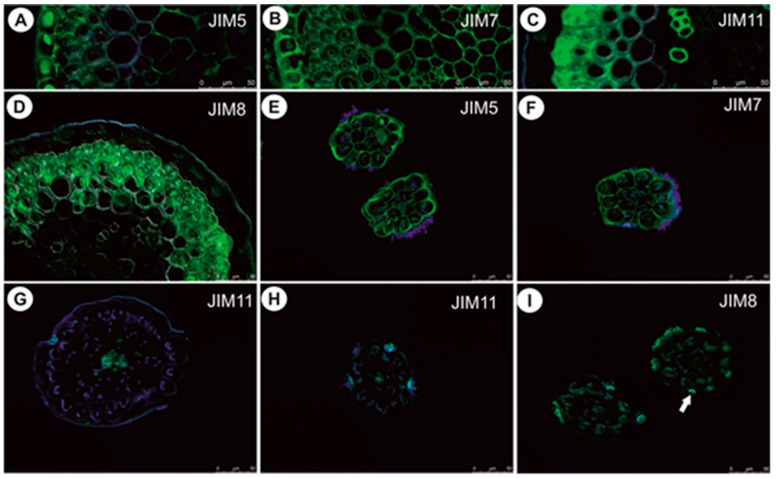
Pectin (JIM5, JIM7), extensin (JIM11) and arabinogalactan protein (JIM8) detection in inflorescence stalk and leaf-like shoots of U. neottioides. (**A**) Presence of the JIM5 in inflorescence stalk, bar 50 µm. (**B**) Presence of the JIM7 in inflorescence stalk, bar 50 µm. (**C**) Presence of the JIM11 in inflorescence stalk, bar 50 µm. (**D**) Presence of the JIM8 in inflorescence stalk, bar 50 µm. (**E**) Presence of the JIM5 in leaf-like shoot, bar 50 µm. (**F**) Presence of the JIM7 in leaf-like shoot, bar 50 µm. (**G**) and (H) Presence of the JIM11 in leaf-like shoot, bar 50 µm. (**I**) Presence of the JIM8 in leaf-like shoot; note a positive signal in trichomes (arrows), bar 50 µm.

In anchor stolons, the pectic epitope recognized by the JIM5 and JIM7 antibodies are abundant in primary cell walls of various cell types: epidermis, parenchyma, phloem (Figure 8A–C). They are present in the intercellular matrices (primary cell walls) between sclerenchyma fibers and absent from the fiber secondary cell walls (Figure 8B). The JIM5 epitope is detected in the interior of fiber cells (Figure 8B). Both JIM5 and JIM7 epitopes also occur in vacuolar material in epidermal cells (Figure 8A–C). Extensin epitope (JIM11) is in the intercellular matrices (primary cell walls) between sclerenchyma fibers and the fiber secondary cell walls and is also detected in cell walls of vessels (Figure 8D,E). It occurs in cell walls of barrier cells of trichomes (Figure 8E). AGPs recognized by JIM8 are detected inside sclerenchyma fiber cells and inside trichome cells (Figure 8F). In the basal portion of inflorescence stalks, the occurrence of pectic epitopes recognized by the JIM5 (Figure 9A) and JIM7 antibodies (Figure 9B), the extensin epitope (JIM11) (Figure 9C) and the arabinogalactan protein (AGP, JIM8) (Figure 9D) is similar to that in anchor stolons. In filamentous leaf-like shoots, pectic epitopes recognized by the JIM7 and JIM5 antibodies are abundant in the primary cell walls of various cell types (Figure 9E,F). The extensin epitope (JIM11) is abundantly present in intercellular spaces of parenchyma cells, which surround the vascular bundle, and also in cells of the vascular bundle (Figure 9G,H). The AGPs recognized by the JIM8 are detected in barrier cells of trichomes and in algae which grow on the organ surface (Figure 9I).

### 2.2. Utricularia reflexa

#### 2.2.1. Shoots

**Figure 10:**
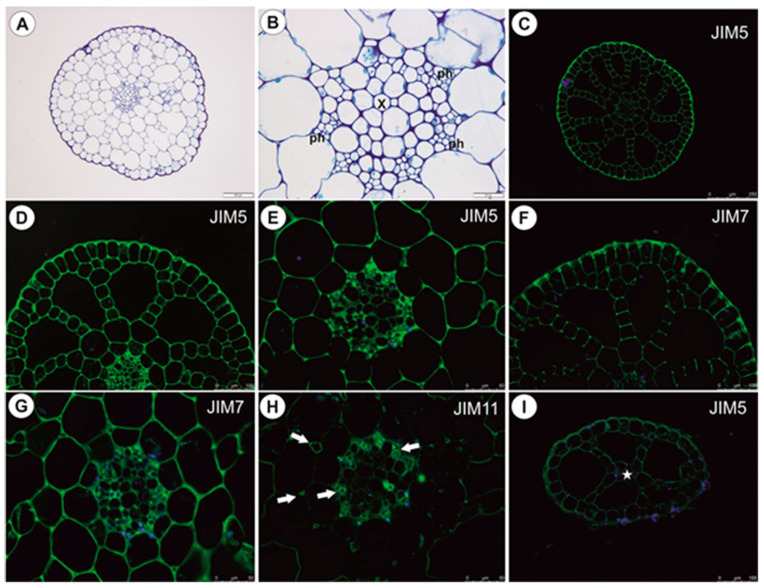
Anatomy and immunochemistry of shoots and leaf-like shoots of *U. reflexa*. (**A**) Transversal shoot section, bar 100 µm. (**B**) Shoot pith anatomy; xylem vessel (x), phloem (ph), bar 20 µm. (**C**–**E**) Presence of the JIM5, bar 250 µm (**C**), 100 µm (**D**) and 50 µm (**E**). (**F**,**G**) Presence of the JIM7, bar 100 µm (**F**) and 50 µm (**G**). (**H**) Presence of the JIM11 (arrows), bar 50 µm. (**I**) Presence of the JIM5 in leaf-like shoot, bar 100 µm.

The small pith is surrounded by a large aerenchymatic cortex and an epidermis (Figure 10A); the pith (cylinder) is ectophloic. The vessel is located almost centrally and is surrounded by large parenchyma cells with thick cell walls (Figure 10B). The pectic epitope recognized by the JIM5 antibody is abundantly present in the walls of various cell types (Figure 10C–E) and the occurrence of pectic epitope recognized by the JIM7 antibody is similar to that by the JIM5. A closer inspection of the pith revealed that a larger amount of this epitope was found in thick cell walls of parenchyma cells (Figure 10G) in comparison to the JIM5 (Figure 10E). The extensin epitope (JIM11) is abundantly present in intercellular spaces of parenchyma cells and in phloem cells (Figure 10H).

#### 2.2.2. Leaf-Like Shoots

These have a very simple structure. The single vascular bundle is surrounded by a one-layered aerenchyma and an epidermis (Figure 10I). The occurrence of pectic epitopes recognized by the JIM5 and JIM7 antibodes (Figure 10I) is similar to that in the main shoot.

## 3. Discussion

In *U. neottioides*, two shoot systems can be distinguished: organs (stolons, inflorescence stalk), which possess sclerenchyma and so are resistant to water current, and organs without sclerenchyma (leaf-like shoots), which are submissive to the movement of water. In contrast with typical aquatic *Utricularia* species from the section *Utricularia*, which grow in standing or very slowly streaming waters, the morphology and anatomy of *U. neottioides* are highly specialized. In *U. reflexa* (our results), *U. vulgaris* L. [15], *U. stygia* Thor [16,17], *U. bremii* Heer, *U. intermedia* Hayne, *U. minor* L., *U. ochroleuca* Hartm., [16] and *U. breviscapa* Wright ex Griseb. [17], submerged shoots have very well-developed aerenchyma, however, there is a lack of sclerenchyma. These organs also have a symmetric structure. *Utricularia neottioides* stolons (anchor and running) are asymmetric (dorsiventral asymmetry, but also show asymmetry in cell size), have only small intercellular spaces, but have sclerenchyma. Luetzelburg [18] also observed sclerenchyma (“Steinzellen”) in the stolons (“rhizomastes”; his Figure 34) of *U. neottioides*, however, our results show another distribution of the sclerenchyma cells and variations in their occurrence. Sclerenchyma in anchor stolons may have a protective function against mechanical injury by the water current and by material transported by water but may also protect these organs against grazing by animals. Generally, sclerenchyma enables plant organs to withstand various mechanical strains, which may result from stretching, bending, weight and pressure [19]. We showed that sclerenchyma cells in *U. neottioides* organs retained their protoplasts up to maturity, so their cell walls may continue to be modified in response to external stress factors.

In Podostemaceae, which occur in similar habitats to *U. neottioides*, silica bodies are present in long-lived parts such as roots and stems. These may perform a similar function to the sclerenchyma in *U. neottioides* stolons [20,21]. According to these authors, silica bodies may also prevent the plant from collapsing during short periods of drought. Anchor stolons in *U. neottioides* have a thick cuticle in the epidermal cells and the cell walls of some epidermal cells show lignification: both characteristics strengthen the structure of these organs. Lloyd [22] found similarities between *U. rigida*, *U. neottioides* and Podostemaceae. Van Steenis [7] classified rheophytic *Utricularia* and Podostemaceae, which both grow in turbulent waters, to the same group–lepidorhephytes. Rutishauser [23] compared anchor stolons of rheophytic *Utricularia* to the holdfasts in Podostemaceae, Hydrostachyaceae and seagrasses (see section on *Saxicolella amicorum* root, Figure 4 in Ameka et al. [24], which resembles the anatomy of the anchor stolon of *U. neottioides*). Adhesive trichomes along the lower (ventral) side of anchoring organs occur both in rheophytic *Utricularia* and Podostemaceae as a functional convergence. 

Adhesive trichomes were described in Podostemaceae [23]; these trichomes are reported to secrete superglue-like substance, which affixes plants to the rock surface. However, some authors noted that sticky extracellular polymeric substances produced by cyanobacteria are essential for anchoring of these plants [25]. We noted that secretion produced by adhesive trichomes of *U. neottioides* stain positively with MB/AII. Additionally, microorganisms are attached to this secretion. The role of cyanobacteria biofilms for *U. neottioides* anchoring requires further testing, as does the composition of the adhesive trichome secretions. The adhesive plant secretion may have lipid or pectic character. For example, in parasitic plants, an adhesive epithelium occurs and secretes a lipidic glue (*Viscum*) or pectin-rich polysaccharides (*Cuscuta*, *Cassytha*) to hold the parasite and host together [26].

The shape of the *U. neottioides* anchor stolon system could be compared with anchoring holdfasts of some crinoids (see e.g., [27,28,29]). However, this is only analogy with the anchoring system between a specialized carnivorous plant and specialized animals. We found that *U. neottioides* anchor stolons store nutrients (starch) and mucilage. Stolon cells possess chloroplasts, so these organs perform photosynthesis and can support the leaf-like shoots in this respect. Running stolons produce new rosettes, which can give rise to new plants (vegetative reproduction); it should then be considered that plants growing on one rock may be one clone. Thus, in addition to anchoring, the stolon system of *U. neottioides* can perform multiple other functions.

Stănescu and Toma [15] could not distinguish the phloem elements from the xylem ones in the submerged shoots of *U. vulgaris*. They noted that the central cylinder consisted of a homogeneous mass of polygonal cells with cellulose cell walls. This contrasted with Schweingruber et al. [16], who noted that in submerged shoots of aquatic *Utricularia*, the xylem and phloem within the central cylinder were difficult to distinguish. These authors observed a circularly arranged, solitary unlignified vessel in *U*. *vulgaris*. In other species, there were few to one (*U. intermedia*) isolated unlignified vessels. In contrast, we did not have any problem distinguishing the xylem and phloem in the stolons of both *U. reflexa* and *U. neottioides*. Their vessels have lignified cell walls. In *U. neottioides*, vessel diameters are smaller than those observed in aquatic species from the section *Utricularia* [16].

The basal portion of the inflorescence stalk ([1] used the term peduncle for this organ) of *U. neottioides* has a lignified fiber ring; a similar sclerenchyma ring was observed in the flower stalks of various *Utricularia* species, e.g., [16,30]. Schweingruber et al. [16] noted that in the flower stalks of aquatic *Utricularia*, there was a cortex with well-developed aerenchymatic intercellulars. This is in contrast with *U. neottioides*, where the aerenchyma is lacking in the cortex. This trait possibly provides a more compact and rigid structure for the stalk, which experiences rapid water currents.

The leaf-like shoots of *U. neottioides* have a very simple anatomy, similar to that of *U. reflexa* (our results), *U. aurea* [31], *U. gibba* [32] and *U. stygia* [17], but without forming aerenchyma. These organs of aquatic *Utricularia* species are anatomically simpler when compared to both the large “leaves” of giant-leaved *Utricularia* species such as *U. humboldtii* with bifacial blade and palisade parenchyma [33], and also the small-leaved terrestrial species such as *U. dichotoma* [34], *U. uniflora*, and *U. paulineae* [17]. We observe some differences in the immunodetection of cell wall components between *U. reflexa* and *U. neottioides*. In both species, the pectic epitopes recognized by the JIM5 and JIM7 antibodies are abundant in primary cell walls of various cell types. However, in *U. reflexa*, accumulation of these epitopes occurs in the pith; this is associated with the occurrence of thick-walled parenchyma cells, which are missing in pith of *U. neottioides*. Some differences found are associated with the occurrence of sclerenchyma in the stolons of *U. neottioides*. Epitopes recognized by the JIM5 and JIM7 are absent from the sclerenchyma secondary cell walls. In both species, extensin epitope (JIM11) is connected with pith and vascular tissues, however, besides in *U. neottioides* this epitope occurs also in cell walls of sclerenchyma cells. 

The shoots of aquatic *Utricularia* from the sect. *Utricularia* are covered by small epidermal trichomes, which also occur on short, modified shoots in turions [35]. Trichomes of *U. neottioides* shoots have a similar architecture but their terminal cell is strongly elongated. These trichomes, which were noted incorrectly as unicellular [4], could significantly increase gas exchange surfaces. In line with the adaptation for living in strong currents and with the high density of elongated trichomes found in *U. neottioides*, remarkable ecophysiological characteristics were estimated in this species [4]. The high CO_2_ compensation point of photosynthesis of leaf-like shoots (17.1 µM CO_2_) indicates invariably a ca. 2.5–5 times lower CO_2_ affinity than is usual in other aquatic *Utricularia* species. On the contrary, the aerobic dark respiration rate was extremely high, around 8–12 times higher than is usual in other aquatic *Utricularia* species. Such a high respiration rate might cause plant sensitivity to oxygen shortage in the ambient water and, moreover, it must be counterbalanced by a very high net photosynthetic rate, which requires a very high CO_2_ concentration in the water. Alternatively, the plant may be partly dependent on the uptake of organic substances from the water (by the trichomes) or on the extensive uptake of organic substances from carnivory (by traps). Further research should specify the relative importance of elongated trichomes and carnivory for nutrient uptake.

Although some authors [4,36] had doubts regarding the trap occurrence in *U. neottioides*, traps were recorded by several researchers in the past: Luetzelburg (as *U. Herzogii*, his figures: 37–39 [18]) and [1,22]. We have observed them in plants from populations from Minas Gerais as well. Traps were also recorded in populations in Sao Paulo, Goias, and Rio de Janeiro States (S. Silva, V. Miranda, unpublished data).

## 4. Materials and Methods

### 4.1. Plant Material

Material of *Utricularia neottioides* was collected from small streams near Delfinópolis, Serra da Canastra region, Minas Gerais State, Southeastern Brazil (Figure 1A–C). The pH of the streams inhabited by *U. neottioides* ranged from 5.0 to 6.4 (pH meter Tri-Meter EC-983; see Table 1). Material of *U. reflexa* (from Okavango Swamp, Botswana) was taken from the collection at the Institute of Botany CAS at Třeboň, Czech Republic. The plants were grown in 3 L miniaquaria and sedge litter was used as substrate to create a dystrophic environment. The poorly branched plants were 20–30 cm long.

### 4.2. Morphological and Anatomical Studies

Material of both *Utricularia* species was fixed and later processed as in Lustofin et al. [37]: materials were fixed in a mixture of 2.5% or 5% glutaraldehyde with 2.5% formaldehyde in a 0.05-M cacodylate buffer (Sigma-Aldrich, Sigma-Aldrich Sp. z o.o. Poznan, Poland; pH 7.2) for several days, washed three times in a 0.1-M sodium cacodylate buffer and post-fixed in a 1% osmium tetroxide solution at room temperature for 1.5 h. Dehydration using a graded ethanol series, infiltration and embedding using an epoxy embedding medium kit (Honeywell Fluka™, Honeywell Specialty Chemicals Seelze, Seelze Germany) followed. After polymerization at 60 °C, sections for the TEM were cut at 70 nm using a Leica Ultracut UCT ultramicrotome, stained with uranyl acetate and lead citrate. The stolon ultrastructure was analyzed using a Hitachi H500 transmission electron microscope (Hitachi, Tokyo, Japan), at an accelerating voltage of 75 kV and Hitachi UHR FE-SEM SU 8010 at 25 kV, which are housed at the University of Silesia in Katowice. The semi-thin sections (0.9–1.0 µm thick) prepared for the LM were stained with aqueous methylene blue/azure II (MB/AII) for 1–2 min and examined using Olympus BX60 and Nikon Eclipse E400 light microscopes. For the SEM, material was fixed (as above), later dehydrated and dried using supercritical CO_2_. They were then sputter-coated with gold and examined at an accelerating voltage of 20 kV using a Hitachi S-4700 scanning electron microscope, which is housed at the Institute of Geological Sciences, Jagiellonian University in Kraków, Poland. About ten replications of material samples (each organ) were studied.

Histochemical procedures with fixed material using PAS reaction (the periodic acid-Schiff reaction), ruthenium red, and Lugol’s solution were performed to detect the polysaccharides, mucilage, starch grains, and proteins [38]. Sections through *U. neottioides* and *U. reflexa* stolons and shoots were analyzed using a polarized light microscope (Carl Zeiss Axio Lab.A1) in the Department of Environmental Analysis, Geological Mapping and Economic Geology; AGH University of Science and Technology. Parts of *U. neottioides* anchor stolons were stained with zinc iodine chloride solution to visualize lignified cell walls of the sclerenchyma according to [39].

### 4.3. Immunodetection of Cell Wall Components

Stolons and shoots were fixed overnight at 4 °C in 8% (*w*/*v*) paraformaldehyde (PFA) with 0.25% (*v*/*v*) glutaraldehyde (GA) in a PIPES buffer [38]. Plant material was then embedded in Steedman’s wax and sectioned. Rehydrated sections were blocked with a 1% BSA in PBS buffer and incubated with primary antibodies against pectins (JIM5 and JM7), arabinogalactan (JIM8), and extensin n (JIM11) overnight at 4 °C (see [40,41] and literature cited therein; rat monoclonal antibody JIM5, JIM7, JIM8, JIM11, and JIM13 was obtained from Plant Probes, UK) and secondary antibody goat anti-rat conjugated with FITC (Abcam: Abcam plc, registered in England and Wales with Company Number 03509322, Discovery Drive, Cambridge Biomedical Campus, Cambridge, CB2 0AX, UK). Chromatin in the nuclei was stained with 7 µg/mL DAPI and samples cover-slipped using Mowiol medium. They were viewed with Leica DM6000 B using FITC and DAPI filter combined with DIC (Nomarski contrast). At least 2 different replications were performed for each organ and about 10–20 sections were analyzed from each organ for each antibody used. Negative controls were performed by omitting the primary antibody step, obtaining no fluorescence signal in each control frame for all slides stained (Supplementary Materials Figures S3–S5).

## 5. Conclusions

Due to life in a turbulent habitat, *U. neottioides* evolved specific characteristics including an anchor system with stolons, which have an asymmetric structure, sclerenchyma, and adhesive trichomes on the ventral side. This anchor stolon system performs multiple functions including photosynthesis, nutrient storage, and vegetative reproduction as well as anchorage. In contrast with typical aquatic *Utricularia* species from the section *Utricularia*, *U. reflexa*, which grows freely in standing or very slowly streaming waters and forms monomorphic shoots, *U. neottioides* has a well-developed sclerenchyma system but lacks large aerenchyma. The plants produce traps and *U. neottioides* should be thus treated as carnivorous plant.

## Figures and Tables

**Table 1:** pH and water temperature from *Utricularia neottioides* habitat in Delfinópolis-MG.

pH Range (*n* = 3)	Temp (°C)	Date-Time
4.99–5.02	29.8	07 February 2019–15:15
6.22–6.36	27.1	07 February 2019–17:52
5.66–5.75	26.8	09 February 2019–14:44

## Supplementary Materials

Supplementary materials can be found at https://www.mdpi.com/1422-0067/21/12/4474/s1.
