# Supplementary material for: Life in the Current: Anatomy and Morphology of Utricularia neottioides
Source: Int J Mol Sci. 2020 Jun 23;21(12):4474. doi: 10.3390/ijms21124474 (PMC7352640; doi:10.3390/ijms21124474)
Supplement: Supplementary file 1 [file ijms-21-04474-s001.pdf]

International Journal of
Molecular Sciences

Supplementary Materials: Supplementary materials can be found at www.mdpi.com/xxx/s1.

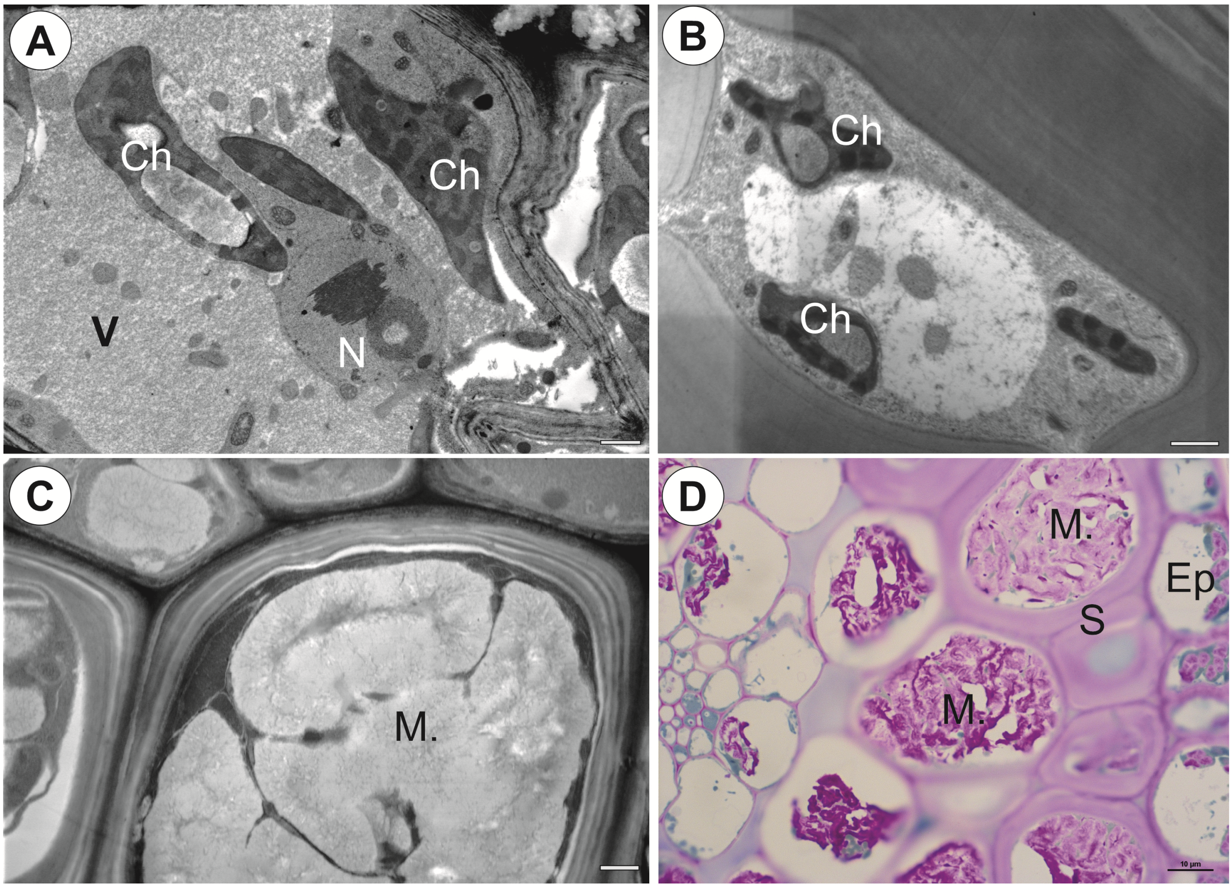

Supplementary Materials Figure S1. *U. neottioides Ultrastructure and histochemistry of cells of anchor stolons.* (A) Ultrastructure of epidermal cells: chloroplast (Ch), nucleus (N), vacuole with material (V), bar 2 µm. (B) Ultrastructure of sclerenchyma cell: chloroplast (Ch), bar 2 µm. (C) Ultrastructure of sclerenchyma cells: note mucilage like material in vacuole (M.), bar 2 µm. (D) part of anchor stolon; sclerenchyma cells (S) contain PAS positive material in vacuoles (M.), epidermal cell (Ep), bar 10 µm.

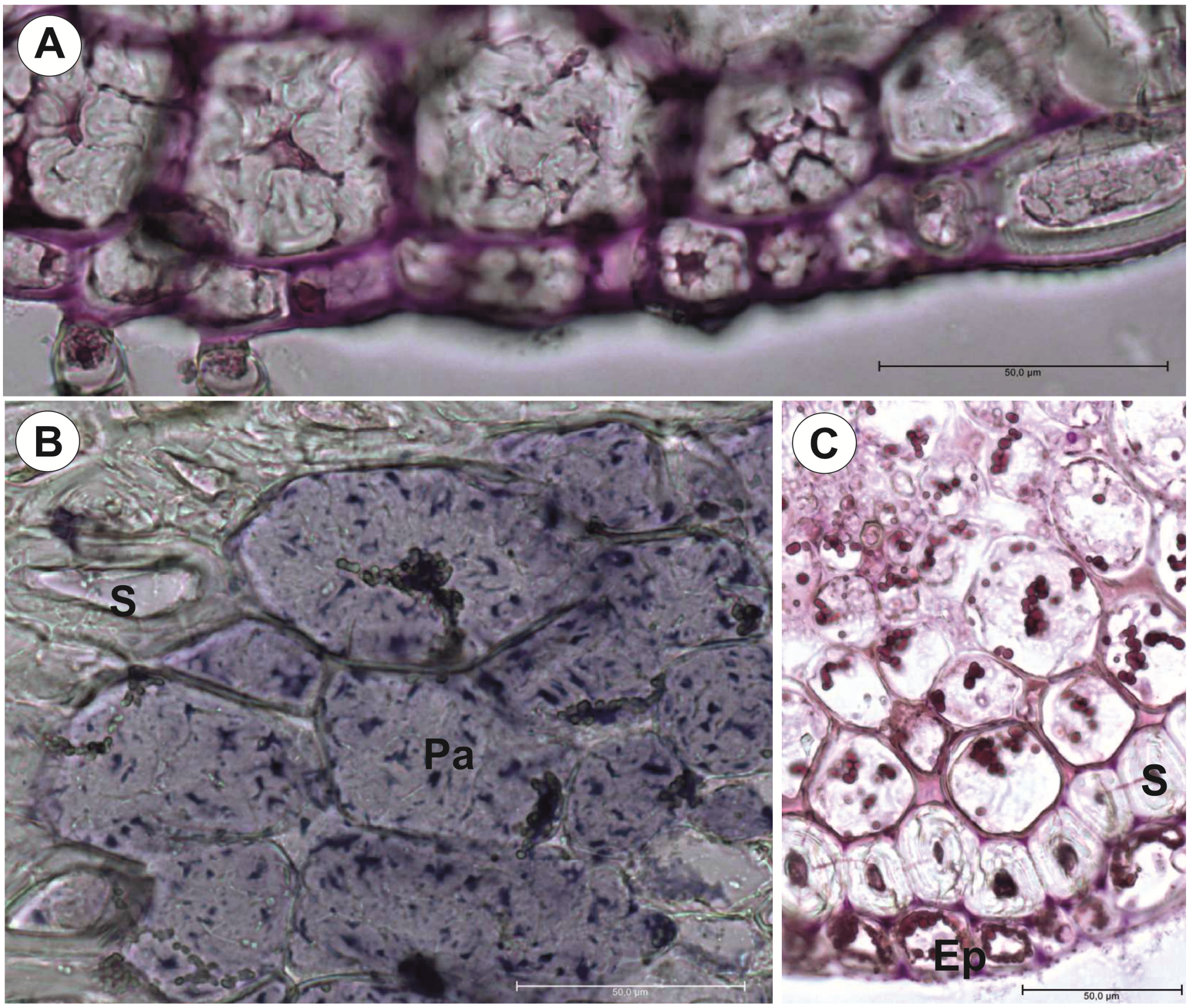

Supplementary Materials Figure S2. *U. neottioides. Histochemistry of cells of anchor stolons.* (A) Ruthenium red staining, (B) and (C) Lugol staining; sclerenchyma cell (S), parenchyma cell (Pa), epidermal cell (Ep). All bars 50μm.

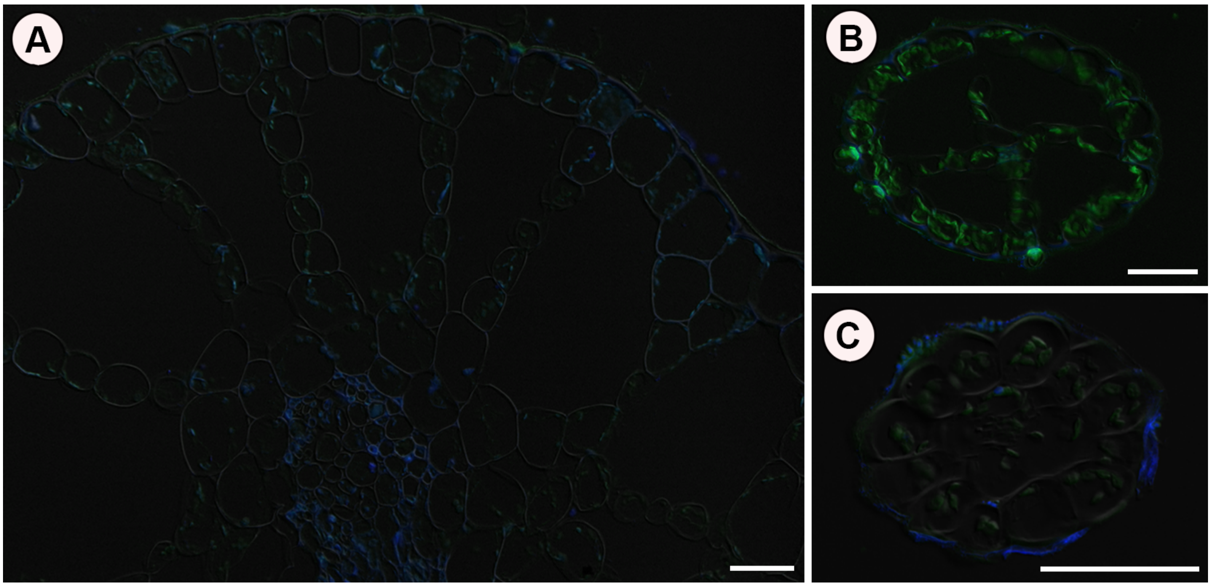

Supplementary Materials Figure S3. *Control reactions of immunolabeling of cell wall components. U. reflexa*: (A) shoot and (B) leaf-like shoot and *U. neottioides*: (C) leaf-like shoot. All bars 50μm

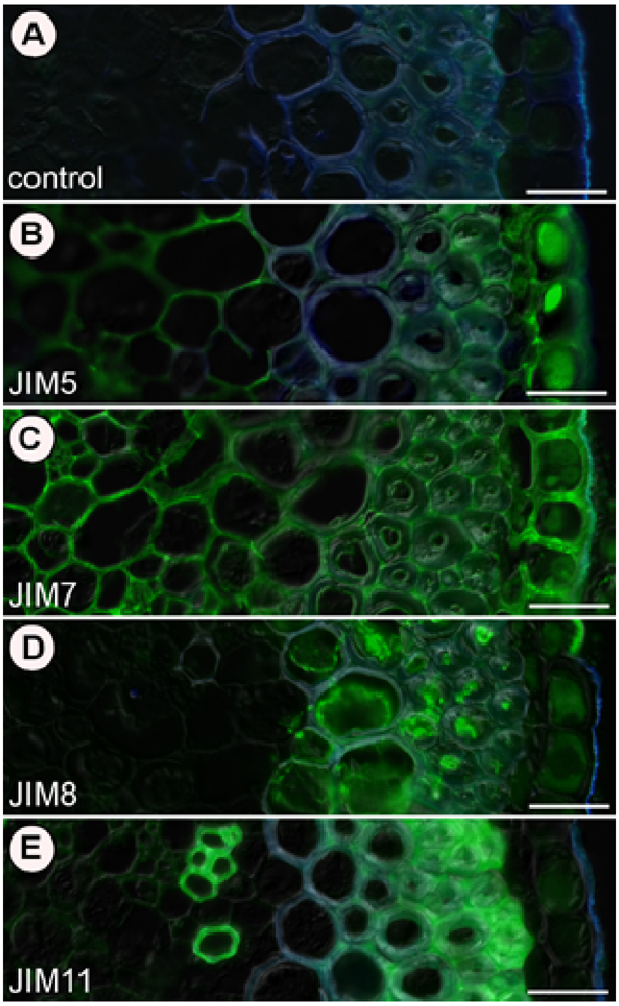

Supplementary Materials Figure S4. *Control reactions and immunolabeling of cell wall components of U. neottioides inflorescence stalk*: (A) control reaction, (B) presence of JIM5, (C) presence of JIM7, (D) presence of JIM8 and (E) presence of JIM11 signal. All bars 50μm

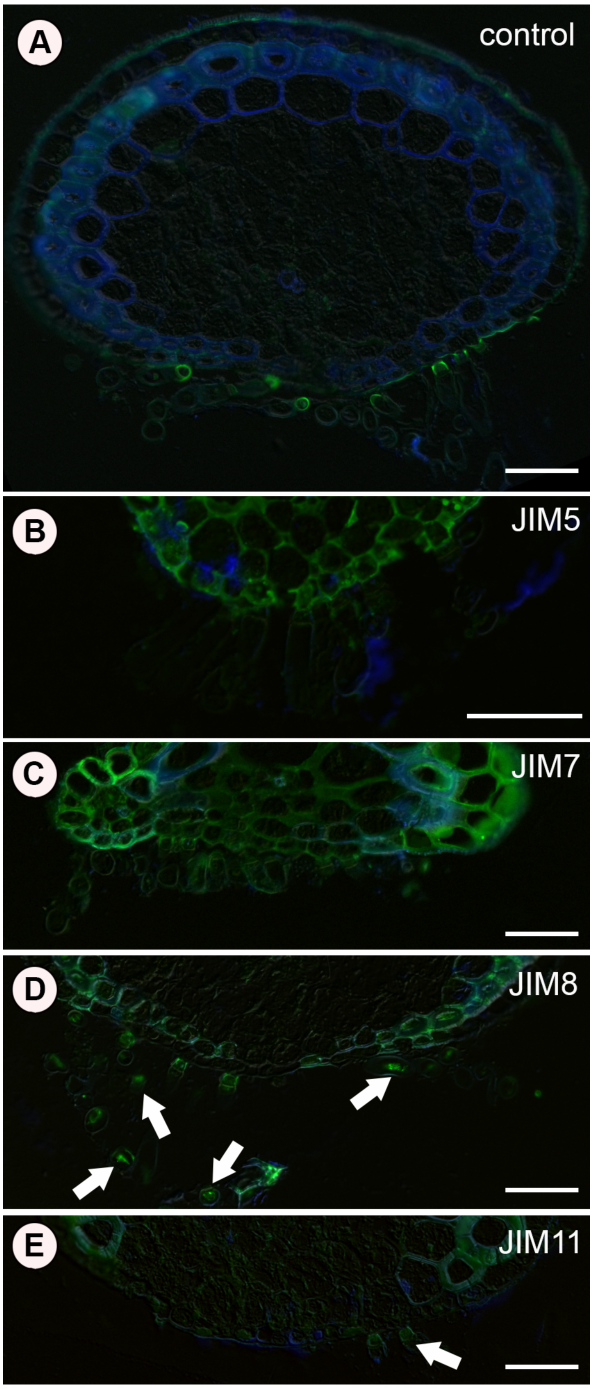

Supplementary Materials Figure S5. *Control reactions and immunolabeling of cell wall components of U. neottioides anchor stolons:* (A) control reaction, (B) presence of JIM5, (C) presence of JIM7, (D) presence of JIM8 and (E) presence of JIM11 signal. Arrows show signal inside trichomes. All bars 100 μm
